# Mechanical and Pore Properties of Foam Concrete Under Salt Erosion Environment

**DOI:** 10.3390/ma18122810

**Published:** 2025-06-15

**Authors:** Weihong Huang, Jiankun Liu, Qinyuan Shi, Weiwei Niu

**Affiliations:** 1 School of Civil Engineering, Sun Yat-Sen University, Zhuhai 519082, China; huangwh76@mail2.sysu.edu.cn (W.H.); niuww3@mail2.sysu.edu.cn (W.N.); 2 Southern Marine Science and Engineering Guangdong Laboratory (Zhuhai), Zhuhai 519082, China; 3 Zhuhai Dahengqin New Urban Center Development Co., Ltd., Zhuhai 519000, China; 13088968471@163.com

**Keywords:** foamed concrete, macro- and micro-properties, compressive strength, pore structure, hydration products, saline environment

## Abstract

This study investigates the evolution of the macro- and micro-scale properties of foamed concrete under different saline environments, including sulfate, chloride, and composite salt conditions. The research focuses on the changes in compressive strength, pore structure, and hydration products of the material. Through full-immersion tests and compressive strength measurements, combined with microstructural characterization techniques such as mercury intrusion porosimetry (MIP) and thermogravimetric analysis (TG), the deterioration mechanisms of foamed concrete under salt attack are systematically explored. The results indicate that Sulfate ions exhibit the most aggressive erosion effect, and the presence of chloride ions can produce a “passivation” effect which partially mitigates the damage caused by sulfate ions. Moreover, increasing the material density and incorporation of mineral admixtures contributes to pore structure refinement, significantly enhancing resistance to salt attack. These findings provide a theoretical basis for the practical application of foamed concrete under a complex salt erosion environment.

## 1. Introduction

Foamed concrete (FC) has been widely used in filling works, ground improvement, structures in high altitudes, and underground structures in recent years due to its low-density, high water absorption, good thermal insulation properties, and ease of construction [1,2,3,4]. These characteristics make FC particularly suitable for application in soft soil regions and complex geological conditions. In this context, its use is especially relevant in Guangdong Province, where soft soil is extensively distributed [5]. However, FC generally has a high porosity and water absorption capacity, with an average water absorption rate approximately 2.5 times that of ordinary mortar, making it highly susceptible to the ingress of groundwater and aggressive ions during its service life [6,7].

In persistently humid environments, expansive calcium compounds can easily form within FC, leading to volumetric expansion, crack propagation, and degradation of mechanical properties [8,9]. These processes significantly reduce the overall mechanical strength and durability of the structure. In engineering environments, water from sources such as soil, groundwater, seawater, and industrial wastewater often serves as the main carrier for aggressive ions such as SO_4_^2−^ and Cl^−^, posing a serious threat to FC used for structural purposes [10].

Among these ions, sulfates are particularly harmful due to their widespread presence in soil, groundwater, seawater, and industrial effluents, and they pose a continuous threat to FC components in underground structures [11,12]. Studies have shown that sulfate attack involves the formation of gypsum and ettringite as well as the degradation of C-S-H gel [13,14]. However, due to the coupled effects of multiple factors including material composition, water-to-cement ratio, ion concentration, and exposure duration, the microscopic deterioration mechanisms remain insufficiently understood.

In addition, unintentional coupling of chloride and sulfate ions frequently occurs in coastal regions and inland areas with salt-rich soils. In such environments, the coexistence of chloride and sulfate ions not only accelerates the corrosion of steel reinforcement but also exacerbates matrix degradation and softening, leading to a significant decline in structural durability.

Although the effects of chloride and sulfate ions on FC have been studied separately [15], current research does not clearly assess how chloride ions influence the expansion and strength reduction caused by sulfate attack in cementitious materials. The resistance of FC to corrosion under combined chloride–sulfate conditions remains insufficiently understood, particularly regarding the performance evolution of FC with different densities under both short-term and long-term exposure. Therefore, it is essential to reveal the underlying mechanisms governing the degradation of mechanical properties and the evolution of pore characteristics in FC under both single and combined salt attacks. A better understanding of how material composition and pore structure influence salt resistance will provide theoretical support and practical guidance for the reliable use of FC in underground structures and complex environmental conditions.

## 2. Methodology

### 2.1. Test Materials

In traditional foamed concrete (FC) systems, cement serves as the primary binder. Commonly used cement types include ordinary Portland cement, rapid-hardening Portland cement, sulfoaluminate cement, and high-alumina cement, with the cement content accounting for 25% to 100% of the total binder material [16,17]. In addition, mineral admixtures such as silica fume, fly ash, lime, incinerator bottom ash, and fly ash ceramsite are frequently used to replace 10% to 75% of the cement content [18,19,20,21,22,23]. These admixtures can improve paste density, enhance long-term strength, and significantly reduce production costs.

In this study, FC was prepared using cementitious materials, a foaming agent, and ground granulated blast furnace slag (BFS) as key components. Mix proportions were optimized through orthogonal experiments. Portland cement (PC) and BFS were used as the main binders. To improve the performance of the BFS-based binder, hydrated lime (HL) was added in an amount equal to one-ninth of the BFS content.

The cement used in the study was composite Portland cement (P.C 42.5), produced by the Tianshan Cement Plant in Xinjiang, in accordance with the production requirements of GB 175-2023 [24]. The BFS was obtained from Lingshou in Hebei Province, meeting the specifications for S95-grade slag powder as defined in GB/T 18046-2017 [25]. The hydrated lime (HL) was analytical grade Ca(OH)_2_ with a purity of no less than 95.0%. The fundamental properties of cement and BFS are presented in Table 1. The TY-type composite foaming agent, provided by Nanjing Daye Building Energy-Saving Technology Co., Ltd. (Nanjing, China), was diluted with water during testing to produce air foam.

### 2.2. Sample Preparation

**Figure 1:**
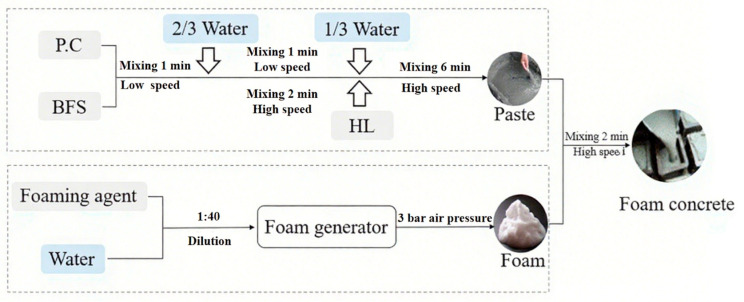
Flowchart of Foamed Concrete preparation.

The study uses foamed concrete with 30% slag powder content (SFC30) for experimentation. The preparation was completed according to the specifications in JGJ/T 341 [26], and the mix design calculations were based on the guidelines of CJJ/T 177-2012 [27]. The preparation process is shown in Figure 1. The foam was produced using a pre-foaming method, where the diluted foaming agent was placed into an air compressor to generate foam. With a fixed water-to-binder ratio (w/b) of 0.5, the foam was used as the main variable. Three foamed concrete samples with different density levels (500, 600, 800 kg/m^3^) were designed, named F1, F2, and F3, with other parameters listed in Table 2.

Simultaneously, the slurry was prepared as follows: the cementitious materials were first poured into the mixing drum to form a uniform dry mix, then 2/3 of the water was added and stirred. Afterward, hydrated lime (HL) and the remaining water were added and mixed thoroughly. Once the slurry was prepared, the pre-produced foam was added, and the mixture was stirred for 2 min before pouring and molding. The samples were then moved to the curing room.

### 2.3. Salt Erosion Test Procedure

Four types of immersion media are set up: clean water (control group), 5% Na_2_SO_4_ solution, 5% NaCl solution, and a composite solution of 5% Na_2_SO_4_ + 5% NaCl. Independent containers are prepared for each salt erosion environment (sealed plastic boxes with dimensions of 575 mm × 390 mm × 190 mm).

**Figure 2:**
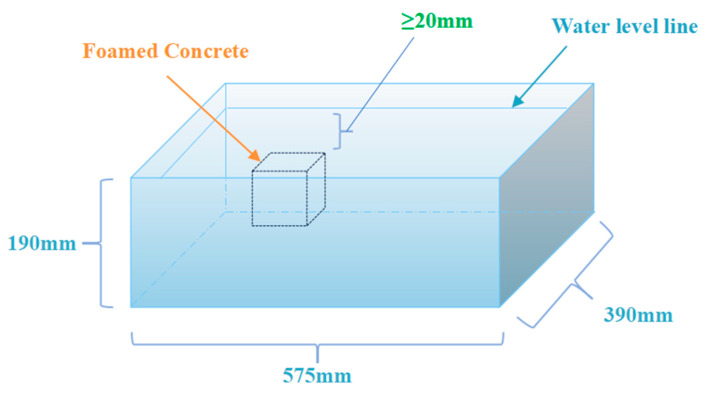
Schematic diagram of full-immersion salt corrosion test for foamed concrete.

Samples with three different densities, F1, F2, and F3, are cured under standard conditions until the specified age (28 days), then demolded and immediately placed in the corresponding salt solution for erosion treatment. Nine specimens (three of each density) are placed in each container, with the liquid level 20 mm above the top of the specimens, as shown in Figure 2, to ensure full immersion. The containers are sealed with lids to prevent evaporation and concentration changes. The erosion periods are set to 28, 56, 90, and 120 days, with samples taken for compressive strength performance and micro-pore characteristics analysis at each age.

### 2.4. Test Methods

#### 2.4.1. Compressive Strength Test

**Figure 3:**
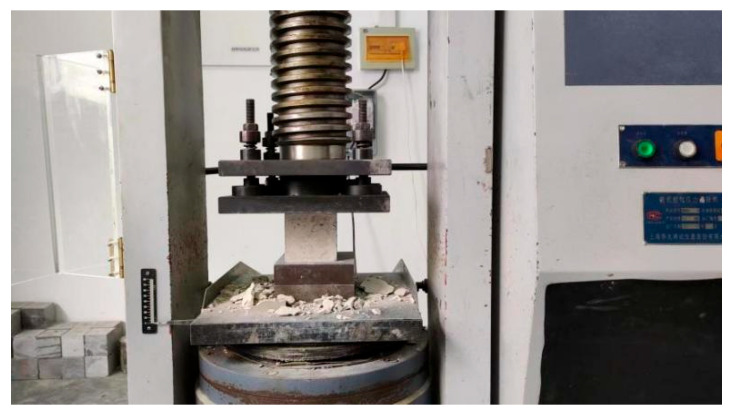
Computer-controlled compression testing machine.

The compressive strength test was conducted in accordance with the “Chinese industry standard Foamed Concrete” (JG/T 266-2011) [28]. For each curing age, three cubic specimens with dimensions of 100 × 100 × 100 mm were selected for each density group to account for randomness in the experimental results. The pressure testing machine is shown in Figure 3.

Before testing, the specimens were dried to constant weight at 65 ± 5 °C. The loading rate was set at 2.4 kN/s. During the loading process, the center of the specimen was aligned with the center of the pressure plate, and the bearing surface was kept perpendicular to the casting surface. To evaluate the material’s resistance to salt corrosion, the corrosion resistance coefficient K_X_ was calculated using Equation (1):

K_X_ = f_X_/f_N_ × 100% (1)

where K_X_ is the corrosion resistance coefficient in X solution; f_X_ is the compressive strength of foamed concrete specimens after exposure to X solution; and f_N_ is the compressive strength of water-cured specimens at the same curing age.

#### 2.4.2. Mercury Intrusion Porosimetry (MIP) Analysis

To monitor the pore characteristics of foamed concrete specimens under various salt corrosion cycles and reveal the evolution of pore structure, a fully automatic mercury intrusion porosimeter (AutoPore V, Micromeritics Instrument Ltd., Norcross, GA, USA) was used to measure the pore size distribution in the range of 0.003–1100 µm. Samples were taken from the core fragments of the crushed specimens after compressive testing, with standard sample dimensions of 10 mm × 10 mm × 10 mm. Prior to testing, the samples were dried in a low-temperature oven for 48 h to avoid measurement errors.

#### 2.4.3. Thermogravimetric Analysis (TGA)

**Figure 4:**
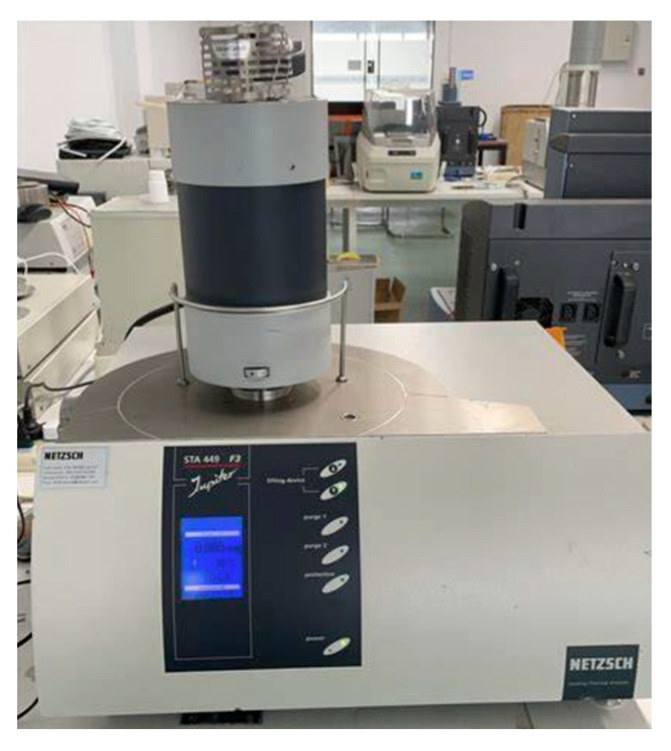
NETZSCH STA 449F3 thermal analyzer.

To investigate the evolution of hydration products during salt corrosion of foamed concrete in different environments, thermogravimetric analysis (TGA) was carried out using a NETZSCH STA 449F3 thermal analyzer (ETZSCH, Selb, Germany) as shown in Figure 4. Standard samples were taken from the core regions, dried, and ground to a particle size below 45 µm. Before testing, the samples were left at room temperature for 60 min, then heated to 1000 °C at a rate of 20 °C/min under a nitrogen atmosphere. The mass loss data and decomposition temperature ranges were recorded.

## 3. Results and Analysis

### 3.1. Effect of Different Salt Environments on the Compressive Strength of FC

**Figure 5:**
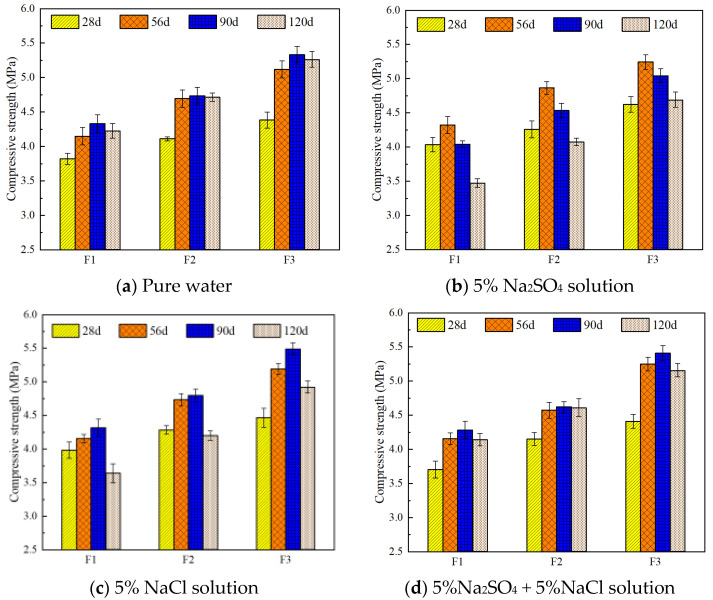
Effect of different saline environments on the compressive strength of FC, F1, F2, and F3 represent specimens with densities of 500, 600, and 800 kg/m^3^.

The compressive strength of foamed concrete under different salt environments is shown in Figure 5, including the strength development of three standard samples (F1, F2, F3) in fresh water, 5% Na_2_SO_4_ solution, 5% NaCl solution, and a combined solution of 5% Na_2_SO_4_ + 5% NaCl.

As seen in Figure 5a,b, the Na_2_SO_4_ solution exhibits a positive effect on the compressive strength of foamed concrete. For example, after 28 days of curing in the sulfate environment, sample F1 shows a slight increase in strength compared to the water-cured control group, with a maximum increase of 5.58% and a minimum of 3.57%. At 56 days, the strength of F1 is 4.21% higher than the water-cured sample. However, when the curing period extends to 90 days, the strength under the Na_2_SO_4_ solution significantly decreases by about 9.1%. At 120 days, the strength of F1 drops by 17.83% compared to the water group, exceeding the 15% compressive strength loss limit defined by JC/T1011-2006 [29] for sulfate-resistant concrete. In contrast, the strength loss of F2 and F3 at 120 days is only 13.60% and 10.87%, respectively, indicating varying degrees of improvement compared to F1. This improvement is likely due to the reaction between Na_2_SO_4_ in the solution and hydration products, which activates the slag powder in the sample and promotes pozzolanic reactions.

In comparison, chloride salt environments have a less significant effect on strength, as shown in Figure 5c. At each curing age, the strength of the samples remains above 95% of the fresh water control group. This suggests that Cl^−^ mainly exists in a free state and only reacts with AFm to form Friedel’s salt, without significantly disturbing the structure. This aligns with the known ability of AFm phases to chemically bind Cl^−^, where sulfate in monosulfate is replaced by Cl^−^ to form Friedel’s salt (C_3_A·CaCl_2_·10H_2_O), releasing SO_4_^2−^ to form ettringite [8]. Furthermore, the solubility of chloride in chloride solution is three times higher than in water, which explains the minimal impact of Cl^−^ salt solutions on the compressive strength of foamed concrete.

Figure 5d shows the compressive strength of foamed concrete in the mixed salt environment (5% Na_2_SO_4_ + 5% NaCl). The trend in strength change is similar, but the degradation appears with a delay. While compressive strength loss occurs in the sulfate environment at 90 days, it is not observed in the composite salt environment until 120 days. This indicates that Cl^−^ can mitigate SO_4_^2−^ attack by reducing ettringite expansion and promoting the formation of stable products. The incorporation of slag powder significantly reduces strength loss in the sulfate environment, attributed to the activation of slag’s latent hydraulic properties, which promotes secondary hydration reactions, enhances matrix densification, and improves corrosion resistance.

### 3.2. Effect of Different Densities on the Salt Corrosion Resistance of FC

**Figure 6:**
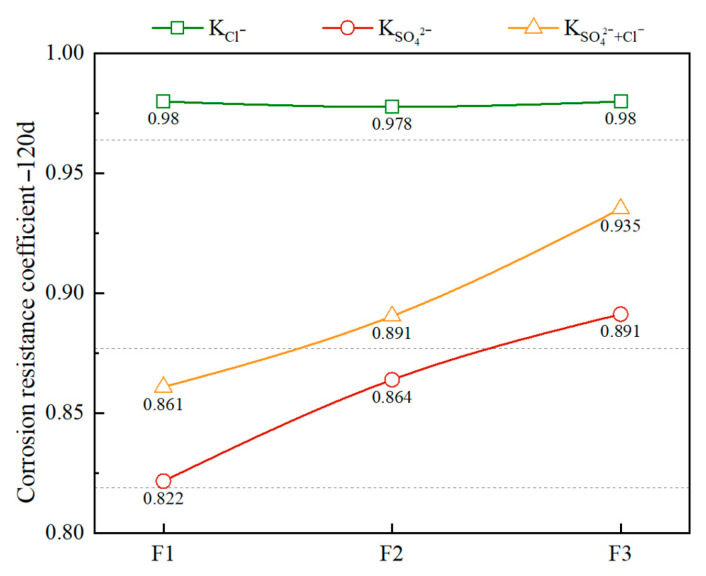
Effect of different densities on the salt corrosion resistance of FC.

The salt resistance coefficients of foamed concrete with different densities after 120 days of salt corrosion are shown in Figure 6. Overall, the resistance of foamed concrete to sulfate (SO_4_^2−^) and composite salt (Cl^−^ + SO_4_^2−^) corrosion increases with increasing density. In the sulfate environment, the sulfate corrosion resistance coefficients of the standard samples F1, F2, and F3 with different density levels are 0.822, 0.864, and 0.891, respectively. In the composite salt environment, the values are 0.861, 0.891, and 0.935, respectively. This confirms that the higher-density samples contain more cementitious materials and aggregates, and have reduced porosity, a denser structure, and thicker bubble walls, which enhance local support capacity and microstructural continuity, thus improving salt corrosion resistance.

In contrast, the resistance of foamed concrete to chloride (Cl^−^) corrosion is less sensitive to changes in density. This may be due to the dominance of closed pores in its structure, where the Cl^−^ transport path is mainly controlled by the connectivity of open pores. The increase in density has a limited effect on improving this connectivity. Moreover, foamed concrete generally contains a low amount of cementitious material and has a developed capillary pore network. Although increasing density reduces porosity, it is difficult to form a sufficiently dense barrier structure. Additionally, microcracks are likely to form during service, providing pathways for Cl^−^ penetration.

In the composite salt environment, due to the mitigating effect of Cl^−^ on the diffusion and reaction processes of SO_4_^2−^, better durability is observed compared to sulfate alone. With the incorporation of blast furnace slag powder, its filling effect and pozzolanic reaction together improve the pore structure and cementitious matrix, thereby promoting the formation and densification of C-S-H gel and enhancing the corrosion resistance of foamed concrete in salt environments.

### 3.3. Effect of Different Salt Corrosion Environments on the Pore Structure Characteristics of FC

#### 3.3.1. Influence of Different Salt Environments on Pore Size Distribution Under Short-Term Salt Exposure (28 Days)

**Figure 7:**
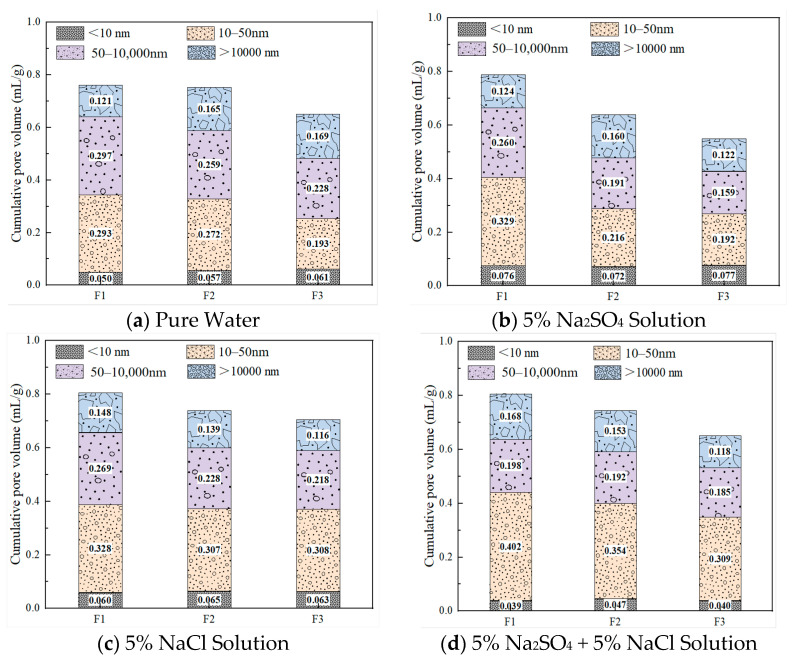
Pore size distribution of FC cured for 28 days under different environments.

The pore structure of FC largely determines its mechanical properties and durability. Figure 7 shows the pore size distribution of foamed concrete with different densities (F1, F2, and F3) after 28 days of curing in four environments: tap water, sulfate, chloride, and composite salt. It is evident that the pore structure undergoes significant evolution under different corrosive environments. Pores are classified by size into: gel pores (<10 nm), medium capillary pores (10–50 nm), large capillary pores (50–10,000 nm), and macrocapillary pores (>10,000 nm) [30]. Gel pores are intrinsic to hydration products, so an increase in their volume fraction is usually regarded as a sign of increased hydration products and thus beneficial to the performance of foamed concrete. Larger pores (>10 μm) are formed due to insufficient compaction between hydration products and negatively affect strength and durability as their size increases.

As shown in Figure 7a, in the tap water environment, the cumulative pore volume of the foamed concrete decreases with increasing density. F3 has the lowest cumulative pore volume, followed by F2 and F1. This is mainly due to the higher solid content in higher-density samples, which leads to stronger paste wrapping and compaction, reducing bubble generation and limiting pore formation space. Additionally, improved paste-filling capability results in a denser pore structure. Although the porosities of F1, F2, and F3 differ, their median pore sizes are similar: F3—10.34 nm, F2—10.41 nm, and F1—10.25 nm. This indicates that their pore size distributions are relatively consistent, likely due to the same foaming system, water-binder ratio, and mixing process used during preparation, which results in a uniform foaming mechanism and stability, thus forming similar pore size characteristics.

In the sulfate environment shown in Figure 7b, large capillary pores significantly decrease. Compared to the tap water environment, the large capillary pore content of F1 to F3 is reduced by 12.58%, 26.32%, and 30.40%, respectively. This is attributed to the activation effect of Na_2_SO_4_ on slag powder, which promotes the formation of hydration products—especially the deposition of ettringite, which fills existing pores, resulting in overall pore refinement and increased gel pore volume. For instance, in F2, the volumes of medium, large, and macro capillary pores decrease from 0.272 mL/g, 0.259 mL/g, and 0.165 mL/g to 0.216 mL/g, 0.191 mL/g, and 0.160 mL/g, respectively. Meanwhile, the gel pore volume increases from 0.057 mL/g to 0.072 mL/g, indicating a more refined pore structure. This is closely related to its moderate porosity and effective ion diffusion pathways. In contrast, the pore refinement effect is less significant in F1 due to loose structure and weak constraint, and in F3 due to restricted diffusion in its denser structure.

In the chloride environment shown in Figure 7c, compared to the tap water environment, the gel pore volumes of F1–F3 increase by 20.00%, 14.04%, and 3.28%, respectively, and the medium capillary pore volumes increase by 11.95%, 12.87%, and 59.59%, while the large capillary pore volumes decrease by 9.43%, 11.97%, and 4.39%, respectively. These changes result from both dissolution and regeneration of ettringite in the slag under chloride action. On one hand, new gel phases are formed; on the other hand, existing large pores transform into smaller pores, thus refining the pore structure.

Additionally, in the composite salt environment, the pore structure development of foamed concrete exhibits a combined effect due to the filler and pozzolanic effects of slag powder. Physically, slag powder acts as a fine filler to occupy voids generated by bubbles and promotes uniform distribution, reducing pore sizes [31]. Chemically, the pozzolanic effect of slag powder consumes calcium hydroxide (CH) and produces dense calcium silicate hydrate (C-S-H) gel, effectively refining and filling the pore structure. As shown in Figure 7d, the medium capillary pores of F1–F3 increase significantly, with increments ranging from 0.082 to 0.116 mL/g.

#### 3.3.2. Evolution of Pore Structure Under Long-Term Salt Exposure (120 Days)

**Figure 8:**
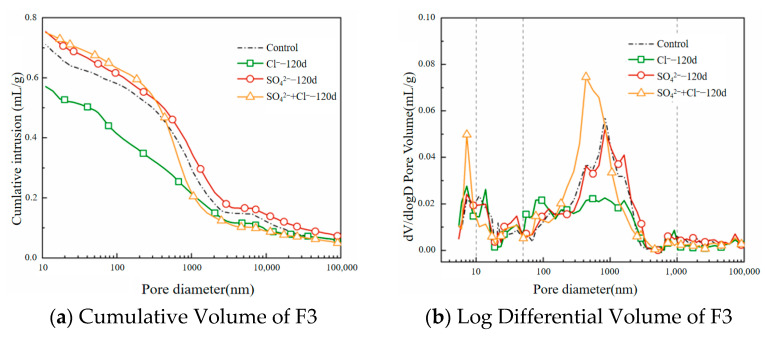
Pore structure evolution of F3 under long-term exposure to sulfate, chloride, and composite salt environments.

Figure 8 illustrates the pore structure evolution trends of F3 foamed concrete specimens (with a density of 800 kg/m^3^) after long-term curing (120 days) in different salt environments. The analysis is based on the cumulative mercury intrusion volume and the distinct peaks in the differential pore volume (dV/dlogD) curves for each environment. Among the various pore sizes, pores larger than 50 nm play a dominant role in determining the material’s permeability and are therefore critical to the ion corrosion resistance of foamed concrete [32].

Taking the pore distribution of F3 after 28 days of curing in tap water as a reference (Figure 8a), F3 exhibits the most favorable pore structure in the chloride salt environment. The total porosity is significantly lower than that of the control group, and the average pore diameter decreases from 52.92 nm to 40.62 nm, representing a 23.24% reduction. This improvement is attributed to the reactive aluminum in the slag, which promotes the formation of Friedel’s salt. As a result, chloride ions become immobilized within the cement-based connected pores, thereby inhibiting the development of large capillary pores while increasing the proportion of gel and medium capillary pores, which enhances material compactness and corrosion resistance.

In contrast, after 120 days of exposure to sulfate and composite salt environments, the porosity of F3 increases significantly, although the underlying mechanisms differ. In the sulfate environment, the excessive formation and precipitation of ettringite within the pores initially helps refine the pore size. However, as these crystals expand and accumulate, gel and medium pores merge and enlarge, ultimately leading to a significant increase in large capillary pores. In the composite salt environment, a more favorable pore structure is observed. The number of large capillary pores falls between those seen in the chloride and sulfate environments, and the gel pore volume fraction is the highest. This suggests that chloride ions exert a suppressive effect on sulfate-induced degradation, effectively slowing down the deterioration of the pore structure. In summary, the chloride environment provides the most beneficial pore structure refinement, while the composite salt environment demonstrates good structural stability under long-term curing. In contrast, the single sulfate environment tends to cause late-stage structural degradation.

### 3.4. Influence of Different Salt Environments on the Phase Composition of FC

#### 3.4.1. Hydration Process Evolution of Different Density Specimens Under Standard Curing (28 Days)

**Figure 9:**
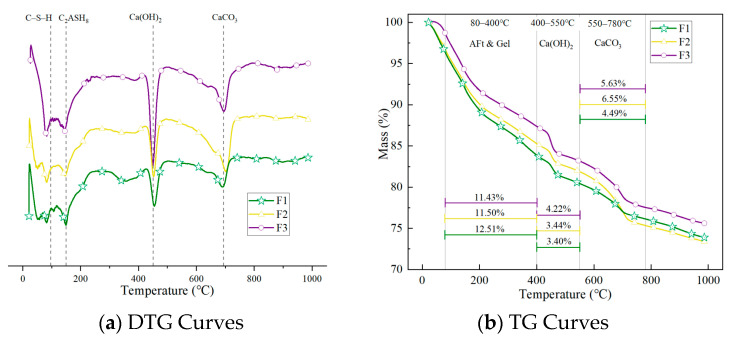
Thermogravimetric results of FC with different densities under standard curing conditions.

Figure 9 presents the thermogravimetric (TG) and derivative thermogravimetric (DTG) curves of foamed concrete with varying densities under standard curing conditions. As shown in Figure 9a, all three sample groups with different densities exhibit distinct mass loss peaks at approximately 97 °C, 150 °C, 455 °C, and 697 °C, corresponding to the decomposition of C-S-H gel, gehlenite hydrate (C_2_ASH_8_), calcium hydroxide (CH), and calcium carbonate (CC), respectively [33].

As the temperature increases, the gel phases in the foamed concrete begin to decompose first. Due to the variety of gels and differences in their structural forms among samples, multiple minor peaks are observed in the 50–400 °C range, indicating a complex mixture of hydration products and suggesting that the hydration process is still ongoing.

In Figure 9a, the decomposition peak of CH crystals appears around 455 °C. The height of this peak clearly reflects the variation in Ca(OH)_2_ formation among different densities: the high-density sample F3 shows the most prominent CH peak, indicating a higher content of cementitious material and a more complete hydration process. In contrast, the low-density sample F1 exhibits a significantly weaker CH peak, due to reduced cementitious content and higher porosity, which limit the generation of Ca(OH)_2_. This suggests that lower density not only decreases the availability of reactive materials but also reduces particle contact efficiency, thus impeding hydration.

Moreover, as Ca(OH)_2_ is a primary alkaline hydration product, it readily reacts with sulfate ions in sulfate environments to form expansive products like ettringite, which can lead to cracking and spalling. A lower amount of Ca(OH)_2_ in the low-density sample reduces the potential for such reactions, which may help slow down the corrosion process and enhance the material’s relative stability under sulfate exposure.

Figure 9b quantitatively reflects the mass loss of various mineral components with rising temperature. In the 80–400 °C range, corresponding to the decomposition of C-S-H gel and free water, the F1 sample shows a mass loss rate of 12.51%, which is significantly higher than that of F2 (11.43%) and F3 (11.50%). This result indicates that F1 contains more volatile components such as adsorbed and capillary water, primarily due to its higher porosity and looser structure. Additionally, the C–S–H gel in F1 tends to have a less compact structure and lower thermal stability, making it more susceptible to thermal decomposition at moderate temperatures and resulting in greater mass loss.

This observation is further supported by mercury intrusion porosimetry (MIP) results, which show that F1 has both higher total porosity and larger average pore diameter. Its loose internal structure provides more channels for water storage and migration, enhancing water evaporation under heat. This characteristic may reduce the high-temperature service stability of the material and imply potential risks when applying low-density foamed concrete in thermal insulation or salt-corrosive environments.

In the 400–550 °C range, corresponding to the dehydroxylation of CH, F1 exhibits a mass loss of only 3.40%, lower than F2 and F3, further confirming its relatively low Ca(OH)_2_ content. The mass loss observed in the 550–780 °C range is primarily due to the decarbonation of CaCO_3_, originating from clinker, mineral admixtures, and carbonation of CH. All three samples exhibit similar trends in this temperature range.

#### 3.4.2. Hydration Process Evolution of Different Density Specimens Under Long-Term Curing (120 Days)

**Figure 10:**
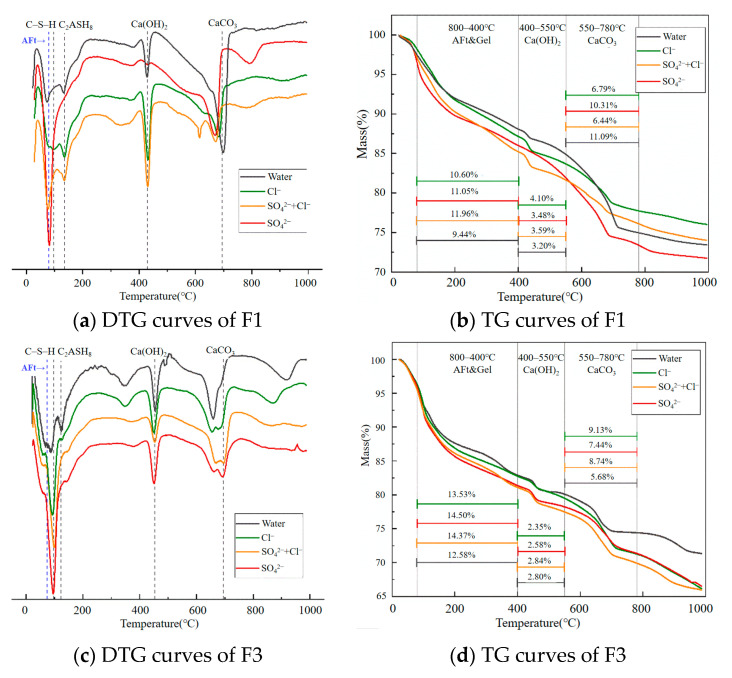
TG curves of F1 and F3 after 120 days of salt exposure.

Based on the DTG and TG results shown in Figure 10, it is evident that NFC specimens cured in saline environments exhibit a distinct peak at around 80.68 °C. As illustrated in Figure 10a, the sulfate environment shows the most pronounced peak at this temperature, followed by the composite salt environment. The Cl^−^ environment exhibits a more moderate peak, while the peak in the freshwater environment is barely noticeable. These features are attributed to the formation of ettringite induced by SO_4_^2−^ and Cl^−^ salts.

Meanwhile, the mass loss of low-density foamed concrete F1 in the 80–400 °C range under different environments is as follows: 9.44% in freshwater, 10.60% in chloride, 11.05% in sulfate, and 11.96% in composite salt environments. It is clear that mass loss under saline conditions is significantly greater than under freshwater, indicating intensified decomposition of hydration products.

High-density foamed concrete F3 also shows evidence of ettringite formation; however, the amount is relatively low. This is reflected by a slight disturbance in the DTG curve at 80.68 °C, as shown in Figure 10c. Figure 10d further reveals that, within the 80–400 °C range, F3 undergoes greater mass loss than F1 across all environments. However, the mass loss attributed to calcium hydroxide (CH) is lower in F3 compared to F1.

## 4. Conclusions

This study explored the evolution of macroscopic and microscopic properties of green foamed concrete with different densities in different salt environments, including sulfate, chloride, and composite salt conditions, focusing on the changes in material compressive strength, pore structure, and hydration products. The main research results are as follows:

(1) This study shows that foamed concrete’s degradation under salt attack follows the sequence sulfate > composite salt > chloride, with sulfate ions causing the most severe deterioration. In mixed Na_2_SO_4_–NaCl solutions, chloride induces a modest passivation effect that retards sulfate corrosion. Moreover, increasing dry density markedly improves resistance: after 120 days in 5 wt % Na_2_SO_4_, strength losses for specimens at 500, 600, and 800 kg/m^3^ were 17.8%, 13.6%, and 10.9%, respectively. Under combined 5 wt % Na_2_SO_4_  +  5 wt % NaCl exposure, low-density samples remained unaffected until 120 days, while high-density samples retained over 95% of their initial strength, confirming density’s protective role.
(2) A comparison between standard curing (28 days) and long-term curing (120 days) results shows that ettringite formed in the early stage temporarily fills pores and seals cracks, thereby reducing porosity. However, as the exposure period extends, the expansive nature of ettringite leads to intensified structural damage. This is especially evident in the composite salt environment, where excessive ettringite formation over time disrupts the original structure, increases pore volume, and induces cracking, ultimately resulting in strength degradation.
(3) Ground granulated blast furnace slag (BFS) contributes to pore structure refinement in foamed concrete through both physical filling and chemical reactions. It acts as a fine aggregate to fill voids and enhance structural compactness. Moreover, the higher the dry density of the material, the more pronounced this refining effect becomes, significantly improving resistance to salt erosion.
(4) High-density specimens, due to their adequate cementitious material and aggregate content, exhibit more complete hydration reactions, resulting in a higher volume fraction of solid products and promoting the formation of large amounts of C-S-H gel, thus optimizing pore size distribution. Furthermore, in saline environments, the formation of ettringite in high-density samples is limited, which helps reduce structural expansion and the risk of microcracking caused by salt erosion.

## Figures and Tables

**Table 1:** Characteristics of Cementitious Materials.

Property	PC	BFS
CaO (%)	63.255	45.22
SiO_2_ (%)	22.77	29.845
Al_2_O_3_ (%)	5.115	13.305
Fe_2_O_3_ (%)	4.125	-
MgO (%)	1.665	6.545
SO_3_ (%)	1.25	1.105
Specific surface (m^2^/kg)	350	436
specific gravity (g/cm^3^)	3.15	2.9
Loss on ignition (%)	1.985	0.46

**Table 2:** Mix proportion design of green FC with different densities.

Mix ID	PC (kg/m^3^)	BFS (kg/m^3^)	HL (kg/m^3^)	w/b	Expected Density (kg/m^3^)
F1	467	180	20	0.5	500
F2	560	216	24	0.5	600
F3	747	288	32	0.5	800

## Data Availability

The original contributions presented in this study are included in the article. Further inquiries can be directed to the corresponding author.
